# Exploring sexual myths and influencing factors among Muslim men in Turkey: a cross-sectional analysis

**DOI:** 10.1186/s12610-025-00296-9

**Published:** 2025-12-03

**Authors:** Aysu Yıldız Karaahmet, Fatma Şule Bilgiç, Shahla Shafaati Laleh

**Affiliations:** 1 Faculty of Health Sciences, Department of Midwifery, Biruni University, Istanbul, Turkey; 2 Faculty of Health Sciences, Department of Midwifery, Çanakkale Onsekiz Mart University, Çanakkale, Turkey; 3 Faculty of Medical Science, Department of Midwifery, Urmia Islamic Azad University, Urmia, Iran

**Keywords:** Sexual myths, Male sexuality, Muslim society, Family planning, Sexual education, Sexual attitudes, Gender roles, Mythes sexuels, Sexualité masculine, Société musulmane, Planification familiale, Education sexuelle, Attitudes sexuelles, Rôles de Genre

## Abstract

**Background:**

This study investigates the relationship between belief in sexual myths and socio-demographic variables, family planning behaviors, and sexual value judgments among Muslim men in Turkey. The objective is to understand how traditional and religiously rooted myths shape male sexual perceptions and behaviors within a culturally sensitive context.

**Results:**

The study involved 953 Muslim men from various regions of Turkey, utilizing an anonymous online survey that included the validated Sexual Myths Scale (SMS) and researcher-developed inventories assessing family planning behaviors and sexual values. The findings revealed that men with lower levels of education (mean SMS score of 75.23) and those living in extended families or rural areas exhibited significantly stronger beliefs in sexual myths (*p* < 0.05). Additionally, high levels of myth endorsement (mean SMS score of 71.01 among non-users of family planning methods) were associated with negative attitudes towards premarital sex and a strong emphasis on female virginity prior to marriage, which are core principles rooted in Islamic religious teachings. However, the disapproval of sexual activity post-menopause and rigid gender role beliefs may reflect broader societal or myth-based beliefs rather than being directly derived from religious doctrine. Regression analysis revealed that sexual value judgments were significantly associated with belief in sexual myths (β = − 0.18, *p* < 0.001). Given that higher scores on the sexual prejudice scale reflect less prejudiced, more positive sexual values, this negative coefficient indicates that more negative or prejudiced sexual value judgments (i.e., lower scores) are associated with stronger endorsement of sexual myths.

**Conclusions:**

The results underscore the influence of educational attainment, family structure, and sociocultural environment on men’s beliefs in sexual myths. These findings emphasize the necessity for culturally sensitive and male-oriented sexual education initiatives aimed at addressing misinformation and challenging traditional prejudices. Targeted interventions are crucial for fostering healthier, more informed sexual attitudes in traditionally structured societies like Turkey.

**Supplementary Information:**

The online version contains supplementary material available at 10.1186/s12610-025-00296-9.

## Résumé

**Contexte:**

Cette étude étudie la relation entre croyance aux mythes sexuels et les variables sociodémographiques, les comportements de planification familiale et les jugements de valeur sexuels chez les hommes musulmans en Turquie. L’objectif est de comprendre comment les mythes traditionnels et enracinés dans la religion façonnent les perceptions et comportements sexuels masculins dans un contexte culturellement sensible.

**Résultats:**

L’étude a impliqué 953 hommes musulmans de différentes régions de Turquie, utilisant une enquête anonyme en ligne comprenant l’Echelle validée des Mythes Sexuels (EMS) et des répertoires développés par des chercheurs évaluant les comportements de planification familiale et les valeurs sexuelles. Les résultats ont révélé que les hommes ayant un niveau d’éducation plus faible (score moyen d’EMS de 75,23) et ceux vivant en familles élargies ou en zones rurales affichaient des croyances significativement plus fortes dans les mythes sexuels (*p* < 0,05). De plus, des niveaux élevés d’approbation des mythes (score moyen d’EMS de 71,01 chez les non-utilisateurs de méthodes de planification familiale) étaient associés à des attitudes négatives envers des relations sexuelles avant le mariage et à une forte insistance sur la virginité féminine avant le mariage, principes fondamentaux ancrés dans les enseignements religieux islamiques. Cependant, la désapprobation d’une activité sexuelle après la ménopause et les croyances rigides sur les rôles de genre peuvent refléter des croyances sociales ou mythiques plus larges plutôt que d’être directement issues de la doctrine religieuse. L’analyse de régression a montré que les jugements de valeur sexuels étaient significativement associés à la croyance en des mythes sexuels (β = –0,18, *p* < 0,001). Étant donné que des scores plus élevés sur l’échelle des préjugés sexuels reflètent des valeurs sexuelles moins préjugées et plus positives, ce coefficient négatif indique que des jugements de valeur sexuels plus négatifs ou biaisés (c’est-à-dire des scores plus faibles) sont associés à une approbation plus forte des mythes sexuels.

**Conclusion:**

Les résultats soulignent l’influence du niveau d’éducation, de la structure familiale et de l’environnement socioculturel sur les croyances des hommes dans les mythes sexuels. Ces résultats soulignent la nécessité d’initiatives d’éducation sexuelle culturellement sensibles et orientées vers les hommes, visant à lutter contre la désinformation et à remettre en cause les préjugés traditionnels. Des interventions ciblées sont cruciales pour favoriser des attitudes sexuelles plus saines et plus informées dans des sociétés traditionnellement structurées comme la Turquie.

## Introduction

 Sexuality is a fundamental and instinctive aspect of human life, influenced by a variety of physical, psychological, cultural, and social factors [1]. While many individuals believe that their sexual thoughts, feelings, and behaviors are independently formed, these aspects are, in fact, shaped by various determinants, such as health status, political climate, cultural norms, and religious beliefs [2, 3]. It is essential to distinguish between sexual myths and religious prohibitions. In Islamic jurisprudence, certain sexual practices—such as intercourse during menstruation or fasting—are explicitly prohibited on the basis of religious doctrine and should not be considered sexual myths. Sexual myths, by contrast, are culturally transmitted misconceptions unsupported by either medical evidence or authentic religious sources. Conflating the two may obscure the sociocultural rather than theological origins of misinformation about sexuality [4, 5].

In societies where sexuality remains a taboo subject, sexual knowledge is often transmitted through informal sources such as peers or family, leading to the internalization of misinformation. This situation negatively impacts individuals’ sexual and reproductive health and undermines family planning efforts [6]. One significant consequence of this misinformation is the prevalence of sexual myths—widespread false beliefs about sexuality that persist across generations [7].

Sexual myths can profoundly affect individual well-being, particularly in communities where sexuality is viewed as a source of shame. In Turkish society, the gender roles assigned to men and women continue to influence sexual relationships, often reinforcing harmful expectations. For instance, men are commonly assumed to always be ready for sexual activity, and practices like masturbation are surrounded by misconceptions [2, 6, 7]. These perceptions not only distort sexual experiences but also contribute to psychological distress and relational dissatisfaction. Utilizing the framework of gender role theory allows for an examination of how traditional gender roles shape sexual attitudes and behaviors, thereby illuminating the challenges individuals encounter while navigating sexual relationships [8].

Understanding a society’s approach to sexuality—including myths, beliefs, and the availability of accurate information—is essential for promoting healthy attitudes and behaviors. Factors such as geography, education, traditions, family structure, and peer influence all play critical roles in shaping sexual knowledge and perceptions. While attitudes toward sexuality may vary between cultures, there is also considerable variation within the same cultural context [9, 10].

The Theory of Planned Behavior posits that individual behavior is driven by intentions influenced by attitudes, subjective norms, and perceived control. Utilizing this theory can help researchers investigate how attitudes toward sexuality and family planning affect individuals’ decisions to engage in sexual behaviors and family planning practices [8]. Sexual myths in Turkey have deep historical roots and have been reshaped over time by cultural and religious dynamics. Exploring these myths and the factors that influence them is crucial for improving sexual and reproductive health, as well as overall societal well-being. Incorporating social constructionist perspectives allows scholars to examine how the meanings attached to sexual roles and identities evolve, influencing individual well-being and behaviors within this cultural setting [8]. In this context, the present study aims to examine the relationship between sexual myths and socio-demographic characteristics, family planning attitudes and behaviors, sexual life, and value judgments regarding sexuality among Muslim men living in Turkish society.

### Patients and methods

#### Study design

This cross-sectional study was conducted online across Turkey between April and May 2023. The study was designed, implemented, and reported in accordance with the Strengthening the Reporting of Observational Studies in Epidemiology (STROBE) guidelines [11].

### Population

The sample size was calculated as 325 men using a power analysis based on the correlation coefficient (*r* = 0.18) reported by Barikani et al. (2023) [12]. During the 30-day data collection period, 953 men were reached. A prospective post-hoc analysis confirmed that the sample size was sufficient.


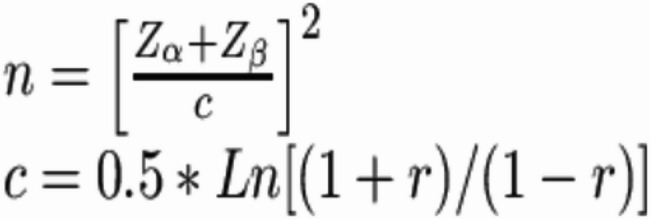


#### Inclusion criteria

Participants were included if they met the following criteria:

- They were of reproductive age between 18 and 64 years.
- They had access to the internet and were able to complete the online form.
- They were actively engaged in sexual activities.
- They voluntarily agreed to participate in the study.

#### Exclusion criteria

Participants were excluded if they met any of the following criteria:

- They had a diagnosed psychiatric illness.
- They had a chronic illness.
- They were diagnosed with a sexually transmitted disease.

### Study outcomes

Data were collected using the following tools:

### Sociodemographic data collection form

This form consisted of six questions regarding participants’ characteristics, including age, education level, and place of residence.

### Family planning attitude and behavior inventory

Developed by the researchers based on relevant literature [8, 13], this inventory consists of seven items related to the use of family planning methods, reasons for preferences, and beliefs about these methods.

### Inventory of sexual value judgments

Also developed by the researchers based on prior studies [6, 13, 14], this inventory contains 16 items that assess participants’ value judgments and religious beliefs regarding sexuality.

Family Planning Attitude and Behavior Inventory and Inventory of Sexual Value Judgments were developed by the researchers specifically for this study. The item generation process began with a comprehensive literature review on men’s reproductive health, family planning, and sexual value systems [6, 8, 13, 14]. An initial pool of 25 and 30 items respectively was created to capture key dimensions such as contraceptive use beliefs, gender roles, sexual responsibility, and moral attitudes toward sexuality. These items were reviewed by a panel of five experts in sexual and reproductive health, psychology, and sociology to assess relevance, clarity, and cultural appropriateness. Based on their feedback, redundant or ambiguous items were removed, resulting in 7 items for the Family Planning Inventory and 16 items for the Sexual Value Judgments Inventory.

Sample items included: “Using modern contraceptive methods may reduce a man’s virility” and “It is a woman’s duty to obey her husband’s sexual requests.” The instruments were pilot tested among 30 men from diverse educational backgrounds to evaluate comprehension and item flow. Content validity was assessed using the Content Validity Index (CVI = 0.87), indicating strong expert agreement. Internal consistency reliability was determined through Cronbach’s alpha coefficients of 0.86 for the Family Planning Attitude and Behavior Inventory and 0.89 for the Inventory of Sexual Value Judgments, indicating acceptable reliability for each scale. These measures met psychometric standards for use in the main survey population (Supplementary Files 1 and 2).

### Sexual Myths scale (SMS)

The factor structure of the Sexual Myths Scale (SMS) was re-evaluated in the present sample of Muslim men to ensure construct validity within this cultural context. A confirmatory factor analysis (CFA) using maximum likelihood estimation supported the original eight-factor structure proposed by Gölbaşı et al. (2016) [14]. Model fit indices indicated an acceptable fit to the data (χ²/df = 2.41, CFI = 0.93, TLI = 0.91, RMSEA = 0.056, SRMR = 0.047). Standardized factor loadings ranged from 0.54 to 0.81 across subdimensions, suggesting that all items contributed adequately to their latent constructs. Measurement invariance across education level (low vs. high) and marital status (married vs. single) was examined using multi-group CFA. Configural and metric invariance were supported (ΔCFI < 0.01), indicating that the SMS measured sexual myths equivalently across these demographic groups. These findings confirm that the instrument retained its factorial validity and cross-group comparability within the male Muslim population.

### Data collection process and bias

A pilot study was conducted prior to data collection, and no revisions to the tools were deemed necessary. Data were collected online from December 1 to December 30, 2023. The first page of the survey outlined the eligibility criteria, and participants who did not meet these criteria were unable to proceed. Participation was voluntary and anonymous. The researchers distributed the online survey to eligible participants through snowball sampling and provided instructions for completion. Informed consent was obtained electronically at the beginning of the questionnaire.

We disseminated the survey link through various channels, including social media platforms, community groups, and online forums. To ensure representation from diverse geographic regions beyond convenience networks, we collaborated with local organizations and community leaders. These efforts included outreach initiatives aimed at rural areas and neighborhoods with lower socio-economic backgrounds. Additionally, we assisted participants with low literacy by providing trained facilitators to help them understand the questions and ensure their responses were recorded accurately.

### Statistical analysis

All analyses were conducted using IBM SPSS version 24.0 (SPSS Inc., Chicago, IL, USA). The Kolmogorov–Smirnov test was employed to assess the normality of the data. Descriptive statistics were calculated, reporting frequency and percentage for categorical variables, and mean ± standard deviation for continuous variables.

To compare Sexual Myths Scale (SMS) scores across groups, independent samples t-tests were utilized. When significant differences were identified among more than two groups, Bonferroni post hoc tests were applied. A significance level of *p* < 0.05 was established.

### Ethical approval

Before data collection commenced, ethical approval was obtained from the Haliç University Clinical Research Ethics Committee (Date: January 25, 2023; Ethics Number: 22). Permission to use the scales was also secured from the respective authors.

During the data collection process via the online questionnaire, participants were provided with necessary information about the study on the first page. If they agreed to participate, they were asked to check the statement “I agree to participate in the study.” It was emphasized that participation was voluntary. Men who completed the online form were considered to have consented to participate in the study. Furthermore, it was clarified that men would not incur any charges or receive any payments for participating in the research.

## Results

A significant difference was found between men’s education levels and both the total score of the SMS and all its sub-dimensions. Additionally, a statistically significant relationship was observed between family type and having children with the SMS total score and all sub-dimensions, except for the sexual orientation and sexual intercourse sub-dimensions. Similarly, significant differences were identified between most sociodemographic variables and the total and sub-dimension scores of the SMS, with the exception of participants’ place of residence and sexual orientation (*p* < 0.05; Table 1). For the Sexual Orientation subscale (by Education Status), the overall effect was significant (see Table 1). Bonferroni-adjusted pairwise tests indicated that higher-education groups had greater scores than at least one lower-education group; the descriptive mean order was 5 > 4 > 6 > 2 > 1 > 3.

**Table 1:** Distribution of sociodemographic variables of men and comparison of SMS and Sub-Dimensions (*n*=953)

Variables	*n*	Sexual Orientation*	Gender*	Age and Sexuality*	Sexual Behavior*	Mastürbation*	Sexual Violence *	Sex *	Sexual Satisfaction*	SMS* Total Puan Average
Education Status										
Not literate ^(1)^	34	11.73±4.66	16.05±6.16	11.11±4.16	7.64±2.98	4.52±2.50	12.64±4.05	5.73±2.62	5.76±1.74	75.23±22.93
Literate^(2)^	146	11.80±4.09	16.97±4.41	11.29± 3.20	8.01±2.22	4.71±2.32	13.77±2.92	5.98±2.24	6.19±1.39	78.75±14.39
Primary school^(3)^	421	11.45±3.83	16.69±4.23	11.38±3.09	7.45±2.09	4.31±2.37	13.51±3.37	6.15±2.22	6.04±1.35	77.00±14.02
Secondary school ^(4)^	11	12.62±1.92	11.25±4.43	7.75±2.43	5.12±1.80	4.00±2.07	8.12±2.85	4.87±1.80	5.00±1.11	58.78±12.11
High school ^(5)^	113	13.03±5.91	10.46±5.12	8.09±3.64	5.04±2.43	4.08±2.03	7.66±4.04	4.79±2.41	4.45±2.01	57.63±20.86
University and up ^(6)^	228	12.20±5.28	10.73±5.43	8.03±3.68	5.17±2.50	3.98±1.92	8.06±4.16	5.20±2.49	4.55±1.78	57.96±20.48
F		2.482	72.063	42.379	52.816	2.302	106.177	9.276	42.705	57.568
p		*0.030*	*0.000*	*0.000*	*0.000*	*0.043*	*0.000*	*0.000*	*0.000*	*0.000*
Bonferroni		5 >4 >6 >2 >1 >3	2>3>1>4>6>5	3>2>1>5>6>4	2>1>3>6>4>5	2>1>3>5>4>6	2>3>1>4>6>5	3>2>1>6>4>5	2>3>1>4>6>5	2>3>1>4>6>5
Family Type										
Nuclear ^(1)^	535	12.00±4.62	14.48±5.61	10.18±3.72	6.73±2.63	4.27±2.20	11.39±4.51	5.73±2.34	5.54±1.74	70.35±19.54
Wide ^(2)^	106	11.56±4.71	14.39±5.69	9.83±3.72	6.44±2.40	4.33±2.09	11.76±4.51	5.58±2.56	5.56±1.74	69.17±19.49
Single^(3)^	312	12.09±4.88	14.22±5.26	8.28±3.46	5.39±2.43	4.09±1.98	8.64±4.47	5.13±2.37	4.66±1.73	59.54±19.44
F		0.677	1.167	3.564	2.299	2.180	2.413	0.198	3.454	2.784
p		0.218	*0.000*	*0.000*	*0.000*	*0.000*	*0.000*	*0.231*	*0.000*	*0.000*
Bonferroni		3>1>2	1>2>3	1>2>3	1>2>3	2>1>3	2>1>3	1>2>3	2>1>3	1>2>3
Marital Status										
Married	641	11.95±4.63	13.96±5.71	9.86± 3.81	6.47±2.62	4.18±2,51	10.99±4.68	5.67±2.42	5.44 ±1.83	68.56±19.98
Single	312	11.79±4.51	15.49± 5.17	10.72±3.48	7.14± 2.46	4.47±2.29	12.38±4.03	5.79±2.31	5.62 ± 1.55	73.43±17.94
t		0.531	−4.134	−3.437	−3.866	−1.937	−4.737	0.724	−1.440	−3.770
p		*0.593*	*0.000*	*0.000*	*0.000*	,*057*	*0.000*	*0.469*	*0.150*	*0.000*
Where to live										
Village ^(1)^	218	11.58±4.27	16.53±4.80	10.86±3.47	7.44±2.33	4.58±2.41	13.15±3.72	5.96±2.30	5.98±1.50	76.11±17.34
County ^(2)^	425	11.91±4.49	15.85±5.10	11.11±3.50	7.26±2.46	4.24±2.20	12.67±3.91	6.00±2.34	5.87±1.62	74.94±17.21
City ^(3)^	310	12.09±4.88	11.22±5.26	8.286±3.46	5.39±2.43	4.09±1.98	8.64±4.47	5.13±2.37	4.66±1.73	59.54±19.44
F		0.808	96.365	65.140	66.844	3.237	112.175	14.085	61.286	80.796
p		0.446	*0.000*	*0.000*	*0.000*	*0.040*	*0.000*	*0.000*	*0.000*	*0.000*
Benferroni		*3>2>1*	*1>2>3*	*2>3>1*	*1>2>3*	*1>2>3*	*1>2>3*	*2>3>1*	*1>2>3*	*1>2>3*
Child presence										
Yes	286	12.01±4.43	15.70±4.85	10.83±3.50	7.31±2.45	4.40±2.21	12.55±3.84	5.85±2.35	5.83±1.63	74.50±17.42
No	667	11.84±4.63	13.97±5.79	9.83±3.76	6.42±2.60	4.21±2.17	11.00±4.69	5.65±2.38	5.36±1.75	68.32±19.99
t		0.647	4.167	3.961	4.999	1.180	5.313	1.198	3.944	4.784
p		0.618	*0.000*	*0.000*	*0.000*	,*000*	,*000*	*0.231*	*0.000*	,*000*

SMS* Sexual Myths Scale, *t* t test, *F* ANOVA Test

Note: Cohen’s d (for t-tests) and eta-squared (η²) (for ANOVA) effect sizes were computed; values ranged from 0.24 to 0.51, indicating small-to-medium practical effects.ANOVA was followed by Bonferroni-adjusted pairwise comparisons. We report the descriptive mean order for readability because Bonferroni does not imply a strict total order; not every adjacent pair is necessarily significant after adjustment. Significant pairwise differences are described in the Results text

Alt Text: Table 1 displaying the sociodemographic characteristics of men (*n*=953) and their comparison across dimensions of the Sexual Myths Scale (SMS). It includes mean ± standard deviation for variables such as education, family type, marital status, living area, child presence, sexual orientation, age, sexual behavior, masturbation, sexual violence, and sexual satisfaction. The table reports F and t values with *p*-values to indicate statistical significance, along with Bonferroni post hoc analysis results. Asterisks denote significant differences (*p* < 0.05)Education status categories: (1) Not literate: unable to read/write; (2) Literate: can read/write, not completed primary education; (3) Primary school: completed primary education; (4) Secondary school: completed secondary education; (5) High school: completed high school; (6) University and up: completed university education or higher

Most men participating in the study reported using a family planning (FP) method, with the withdrawal method being the most commonly preferred option. A significant difference was observed between the FP method used and the total score of SMS, particularly in the sexual orientation and sexual intercourse sub-dimensions. Among men who reported not using FP to avoid pregnancy, significant differences were noted in the sexual orientation and masturbation sub-dimensions. Additionally, the reasons for choosing the withdrawal method showed significant differences in the sexual orientation and masturbation sub-dimensions (*p* < 0.05; Supplementary file 1).

Significant differences were observed in the total score of SMS and its sub-dimensions based on participants’ premarital sexual experiences, beliefs in gender equality in sexuality, and opinions on whether sexual intercourse during menopause is a sin. Statements such as “virginity is important for women,” “men should initiate sexual intercourse,” “a man’s desire must be obeyed,” “society holds specific sexual value judgments regarding sexually transmitted diseases,” and “non-heteronormative identities are sinful” were significantly associated with differences in the SMS total score and nearly all sub-dimensions, except for sexual intercourse and sexual orientation (*p* < 0.05; Supplementary file 2).

Simple regression analysis was conducted to identify factors affecting sexual myths. A significant relationship was found between sexual prejudice scores and SMS total scores (t = − 4.16, *p* < 0.001). In the hierarchical regression model, sexual prejudice remained a significant independent predictor of belief in sexual myths (β = − 0.18, *p* < 0.001; Table 2). Because higher scores on the sexual prejudice scale indicate less prejudiced, more positive sexual values, the negative β coefficient reflects an inverse association: more negative or prejudiced sexual value judgments (i.e., lower scores) were associated with higher levels of belief in sexual myths. Multiple regression analysis controlling for age, education, and family structure demonstrated that sexual value judgments remained a significant independent predictor of belief in sexual myths (β = − 0.18, *p* < 0.001).

**Table 2:** Hierarchical multiple regression analyses predicting sexual Myths scores

Predictor variables	β	t	*p*	*R*²	ΔR²
Model 1: Sexual prejudice	–0.20	–4.16	<0.001	0.052	–
Model 2: Sexual prejudice	–0.18	–3.82	<0.001	0.093	0.041
Age	–0.08	–1.67	0.095		
Education	–0.15	–2.68	0.008		
Family structure	–0.11	–2.16	0.031		

SMS* Sexual Myhts Scale

Note: Dependent variable = Sexual Myths Scale total score; R² and ΔR² refer to explained variance

Alt Text: Table 2 displays the results of hierarchical multiple regression analyses aimed at predicting scores on the Sexual Myths Scale. In Model 1, sexual prejudice is identified as a significant negative predictor of sexual myths scores (β = -0.20, t = -4.16, *p* < 0.001), explaining 5.2% of the variance in the scores (R² = 0.052). In Model 2, which includes additional predictors, sexual prejudice remains significant (β = -0.18, t = -3.82, *p* < 0.001), while age (β = -0.08, t = -1.67, *p* = 0.095), education (β = -0.15, t = -2.68, *p* = 0.008), and family structure (β = -0.11, t = -2.16, *p* = 0.031) are also considered. This model accounts for a total of 9.3% of the variance (R² = 0.093), with an increase of 4.1% from Model 1 (ΔR² = 0.041). These findings suggest that sexual prejudice and educational background significantly influence beliefs about sexual myths

## Discussion

The findings revealed that belief in sexual myths was higher among men with lower education levels, those living in extended family structures, and those residing in rural areas, as well as among individuals using traditional family planning methods. Furthermore, stronger endorsement of sexual myths was associated with men who disapproved of premarital sexual intercourse, placed high importance on female virginity, believed that women should refrain from sexual activity after menopause, regarded different sexual identities as sinful, and thought that men should control the timing and dynamics of sexual intercourse. Regression analysis revealed that stronger sexual value judgments were significantly associated with higher levels of belief in sexual myths. In other words, this sentence indicates a significant positive correlation between the intensity of sexual value judgments and belief in sexual myths.

Sexual myths and limited knowledge about family planning are commonly observed in developing countries, with belief in such myths tending to increase as education levels decrease. Consistent with this trend, the present study found that as men’s education levels declined, their belief in sexual myths correspondingly increased. Additionally, men living in extended families and rural areas were more likely to hold these beliefs. A systematic review of qualitative studies conducted in sub-Saharan African countries also highlighted the widespread presence of sexual myths and misconceptions regarding family planning [15].

Family planning choices significantly influence sexual and reproductive health. However, in many developing societies, traditional beliefs and cultural misconceptions regarding family planning methods remain prevalent [16–18]. These misbeliefs and myths about sexuality can negatively affect individuals’ attitudes toward family planning and the use of modern contraceptive methods [19].

The study found a moderate belief in sexual myths among university students, with male students exhibiting higher levels of belief. Those with lower perceived benefits from interactions with diverse cultures were more likely to endorse these myths. Additionally, intercultural sensitivity emerged as a significant predictor of belief in sexual myths; lower sensitivity correlated with stronger myth endorsement. Enhancing intercultural sensitivity could thus help reduce sexual myths, emphasizing the need for culturally respectful sexual education programs [20]. Another study revealed that Turkish teacher candidates, especially males, held significantly higher beliefs in sexual myths influenced by traditional gender norms. Religiosity was a key factor, as Islamic teachings often restrict open discussions about sexuality. While childhood trauma was not directly correlated with belief in sexual myths, candidates who did not communicate about sexuality with parents showed higher levels of belief. These findings underscore the necessity for educational interventions to foster healthier understandings of sexuality among future educators [5].

Sarpkaya Guder and Tekbas (2022) demonstrated the critical role of sexual health education in reducing beliefs in sexual myths among nursing students. Their study illustrated a significant decrease in sexual myth scores, from 63.65 before the course to 48.64 afterward, indicating the effectiveness of structured educational interventions. Reliance on informal information sources and traditional family structures, which discourage open discussions about sexuality, contributes to the persistence of these myths. Comprehensive sexual health programs are essential for dismantling misconceptions and promoting healthier sexual attitudes among young individuals, enhancing knowledge, and creating a supportive environment for sexual health discussions [21]. Another study showed that intervention programs focused on sexual health education can effectively address and reduce sexual misconceptions and myths among students [22]. Research examining sexual myth endorsement revealed key predictors, including being male, cisgender, heterosexual, younger, more religious, and lacking sex education [23].

In this study, men who believed that the sole purpose of family planning was to prevent childbirth exhibited higher levels of belief in sexual myths. Similarly, those who used traditional methods such as withdrawal and condoms—often chosen for their perceived ease of use—and those who did not use any family planning method at all, were more likely to endorse these myths. While numerous studies have explored attitudes toward family planning and their effects on sexual life [24], few have specifically investigated the impact of sexual myths on family planning-related behaviors and decision-making. This study highlights that sexual myths and family planning attitudes often share similar cultural and cognitive foundations.

Sexuality is a fundamental human instinct that naturally emerges in all individuals [25, 26]. It is a multidimensional concept encompassing gender identity, gender roles, eroticism, sexual orientation, intimacy, pleasure, and reproduction. These dimensions significantly influence people’s thoughts, beliefs, attitudes, desires, and behaviors, even if they are not always openly expressed or consciously experienced [27].

The present study found that men who endorsed traditional and patriarchal beliefs—such as the importance of female virginity, the prohibition of sexual activity before marriage or during menopause, the stigmatization of diverse sexual identities, and the belief that men should control the timing and dynamics of sexual intercourse—were more likely to believe in sexual myths. It is important to distinguish clearly between sexual values rooted in Islamic teachings and culturally transmitted sexual myths. Some questionnaire items—such as discouraging premarital intercourse or the emphasis on female virginity—reflect normative Islamic moral principles and therefore function as religiously grounded sexual ethics rather than myths. These items were included not to label Islamic values as misconceptions, but to capture the wider spectrum of sexual value judgments present within Muslim societies. In contrast, beliefs that lack grounding in Islamic jurisprudence—such as the disapproval of sexual activity after menopause, rigid gender role expectations (e.g., male control over sexual decision-making), or misconceptions regarding women’s sexual pleasure and needs—represent socioculturally transmitted sexual myths shaped by patriarchal norms rather than religious doctrine. Accordingly, in this study, items reflecting Islamic moral teachings were interpreted as value-based attitudes, whereas those without religious support were conceptualized as cultural myths. This distinction ensures that the study does not conflate theological principles with sociocultural misconceptions and provides an accurate cultural framework for interpreting the findings.

Additionally, regression analysis confirmed that as men’s sexual biases increased, their belief in sexual myths also grew. This is consistent with previous research, which has shown that sexual myths are associated with factors such as gender, parental attitudes, premarital sexual experiences, place of residence, and educational background [26, 28, 29]. Consistent with previous validations of the Sexual Myths Scale in mixed-gender Turkish samples [14], the confirmatory factor analysis in this study confirmed the stability of the eight-factor model among Muslim men. The demonstration of measurement invariance across education and marital status strengthens confidence that observed differences reflect true variations in beliefs rather than measurement bias. It should be emphasized that sexual myths differ fundamentally from religious prohibitions in Islam. Certain practices, such as abstaining from sexual intercourse during menstruation, fasting, or pilgrimage, are explicitly regulated by Islamic jurisprudence and stem from theological doctrine rather than cultural misconception. In contrast, sexual myths are socially constructed beliefs that lack scientific or scriptural basis—for example, the idea that masturbation leads to infertility or that women do not require sexual pleasure. The persistence of these myths reflects cultural and patriarchal transmission rather than religious teaching. Therefore, the findings of this study should be interpreted as addressing sociocultural misconceptions rather than doctrinal norms [4, 5]. These results suggest that while sexuality and sexual myths are multifaceted, they intersect around similar socio-cultural characteristics across various groups.

### Limitations of the study

This study has several limitations that must be acknowledged. Firstly, the sample consisted solely of Muslim men living in Turkey, which may limit the generalizability of the findings to other religious or cultural contexts. Data were collected online through self-reported questionnaires, which could introduce social desirability bias, as participants might underreport or misreport sensitive behaviors and beliefs related to sexuality. The exclusion of individuals with a history of sexually transmitted diseases (STDs) may omit an important subgroup whose perspectives could enrich our understanding of sexual myths and related behaviors. Additionally, the study population included participants with varying literacy levels, raising questions about how those with limited literacy completed online questionnaires.

The cross-sectional design of the study restricts the ability to establish causal relationships between variables, capturing only a snapshot in time rather than longitudinal data. Certain topics related to sexuality may be considered taboo in the cultural context, potentially leading participants to provide less than honest responses, thereby affecting the accuracy of the data. Furthermore, restricting participation to individuals with internet access may have limited the sample to specific educational, socio-economic, or age groups, influencing the diversity of the participants. Lastly, while the study provides valuable insights into the relationship between sexual myths and socio-demographic factors, it lacks qualitative data that could deepen the understanding of the underlying reasons for the observed beliefs and attitudes.

## Conclusion

This study found that belief in sexual myths is significantly higher among men with lower education levels, those residing in extended family structures and rural areas, and those using traditional family planning methods. Additionally, men who hold patriarchal views regarding sexuality—such as disapproval of premarital sex, emphasis on female virginity, and beliefs about male dominance in sexual relationships—exhibit stronger endorsement of these myths. The implications of these findings are significant for public health initiatives. Future research should focus on developing and evaluating culturally sensitive sexual education programs targeting populations with lower educational levels and those in rural areas. These programs should aim to dispel sexual myths and promote accurate knowledge about sexuality and reproductive health. Moreover, studies could explore the impact of community-based interventions involving religious leaders, educators, and healthcare professionals in reshaping attitudes toward sexuality. Longitudinal studies are recommended to assess the effectiveness of these interventions over time and to understand the evolving nature of sexual beliefs in different cultural contexts. Employing mixed-methods approaches that integrate online and offline data collection methods, including voice surveys, can help ensure the inclusion of underrepresented groups, such as those with a history of sexually transmitted infections. Finally, expanding research to include diverse populations across various religious and cultural backgrounds will provide a more comprehensive understanding of the prevalence and impact of sexual myths globally, enabling the design of targeted interventions adapted to meet the needs of different communities.

## Supplementary Information

Supplementary Material 1

Supplementary Material 2

Supplementary Material 3

## Data Availability

Data is availabile reasonable request from corresponding author.

## Abbreviations

FP: Family Planning
SMS: Sexual Myths Scale
STROBE: Strengthening the Reporting of Observational Studies in Epidemiology
SD: Standard Deviation
