# Supplementary material for: Exploring sexual myths and influencing factors among Muslim men in Turkey: a cross-sectional analysis
Source: Basic Clin Androl. 2025 Dec 3;35:46. doi: 10.1186/s12610-025-00296-9 (PMC12673704; doi:10.1186/s12610-025-00296-9)
Supplement: Supplementary file 1 — Supplementary Material 1 [file 12610_2025_296_MOESM1_ESM.docx]

**Supplementary file 1. Comparison of the Family Planning Characteristics of Men and the Dimensions of SMS and Sub-Dimensions (n=953)**

| **Variables** | **n** | | **Sexual Orientation*** | **Gender*** | **Age and Sexuality*** | **Sexual Behavior*** | **Mastürbation*** | **Sexual Violence *** | **Sex *** | **Sexual Satisfaction*** | **SMS* Total Puan Average** |
| --- | --- | --- | --- | --- | --- | --- | --- | --- | --- | --- | --- |
| **FP* use case** | | | | | | | | | | | |
| **Yes** | 748 | | 12.04±4.77 | 14.13±5.50 | 9.77±3.85 | 6.67±2.59 | 4.37±2.20 | 10.90±4.37 | 5.56±2.37 | 5.37±1.83 | 68.84±20.13 |
| **No** | 205 | | 11.80±4.45 | 14.71±5.62 | 10.35±3.61 | 6.70±2.58 | 4.21±2.18 | 11.82±4.56 | 5.80±2.38 | 5.57±1.66 | 71.01±18.99 |
| **t** | | | .782 | -1.542 | *-2.346* | -.196 | 1.106 | *-3.064* | -1.551 | -1.728 | -1.662 |
| **p** | | | .414 | .122 | ***.019*** | .845 | .270 | ***.002*** | .121 | .084 | .097 |
| **FP- method used** | | | | | | | | | | | |
| **Prezervatif ^(1)^** | 74 | | 12.20±4.21 | 14.64±5.45 | 10.58±4.37 | 6.79±2.21 | 6.79±2.21 | 4.79±2.15 | 11.23±4.32 | 5.58±2.18 | 71.26±18.11 |
| **Vasectomy ^(2)^** | 34 | | 11.88±4.32 | 14.48±4.98 | 10.11±4.56 | 6.68±2.60 | 6.68±2.60 | 4.25±2.18 | 11.47±4.52 | 5.71±2.38 | 70.13±19.13 |
| **Retraction ^(3)^** | 584 | | 11.95±4.17 | 13.75±4.65 | 10.23±3.76 | 5.65±2.31 | 5.95±2.91 | 4.48±2.45 | 10.53±4.62 | 6.48±2.38 | 70.26±18.43 |
| **Diğer** | 56 | | 11.65±4.78 | 12.65±5.45 | 9.45±4.36 | 5.76±2.40 | 6.36±2.45 | 4.67±2.60 | 11.79±4.12 | 6.19±2.18 | 69.12±19.11 |
| **F** | | | 1.122 | 2.670 | 2.589 | 2.423 | -1.328 | -2.121 | -3.780 | -.462 | -1.714 |
| **p** | | | .262 | ***.000*** | ***.049*** | ***.005*** | ***.000*** | ***.043*** | .064 | *.****005*** | ***.024*** |
| **Bonferroni** | | | **1>3>2>4** | **1>2>3>4** | **1>3>2>4** | **1>2>4>3** | **1>2>4>3** | **1>4>3>2** | **4>2>1>3** | **3>4>2>1** | **1>3>2>4** |
| **Why FP used** | | | | | | | | | | | |
| **Not having children** |  |  |  |  |  |  |  |  |  |  |  |
| **Yes** | 32 | | 10.33±3.69 | 14.00±5.48 | 9.73±3.34 | 6.43±2.43 | 3.26±1.65 | 11.70±5.18 | 5.66±2.18 | 5.20±1.84 | 66.33±19.02 |
| **No** | 921 | | 11.94±4.59 | 14.50±5.58 | 10.14±3.72 | 6.70±2.59 | 4.30±2.19 | 11.46±4.49 | 5.71±5.51 | 5.51±1.73 | 70.29±19.46 |
| **t** | | | *-1.904* | -.492 | -.598 | -.593 | ***-3.333*** | .285 | -.111 | -.970 | -1.099 |
| **p** | | | ***.026*** | .623 | .550 | .577 | ***.001*** | .776 | .920 | .332 | .270 |
| **Comfortable sex life** |  |  |  |  |  |  |  |  |  |  |  |
| **Yes** | 773 | | 11.27±2.75 | 13.23±2.75 | 1056±3.41 | 7.21±2.32 | 4.87±2.45 | 11.69±4.89 | 5.91±3.72 | 5.76±4.49 | 66.65±19.02 |
| **No** | 180 | | 10.54±2.65 | 12.46±3.23 | 10.65±3.65 | 6.89±2.61 | 4.78±2.21 | 11.19±4.61 | 5.64±4.41 | 5.67±2.18 | 70.54±19.46 |
| **t** | | | -1.333 | -.452 | .-446 | -.953 | -1.543 | .349 | -.120 | -1.456 | -1.122 |
| **p** | | | ***.034*** | .540 | .210 | .089 | ***.004*** | .705 | .123 | .954 | .270 |
| **Reason for not using FP** | | | | | | | | | | | |
| **Beliefs** | 436 | | 12.25±5.58 | 16.50±5.50 | 11.25±3.59 | 7.07±2.66 | 4.21±2.04 | 12.00±3.79 | 6.17±2.65 | 5.92±1.56 | 75.39±19.76 |
| **Societal pressure** | 281 | | 11.88±4.54 | 14.43±5.57 | 10.09±3.71 | 6.68±2.58 | 4.27±2.19 | 11.45±4.53 | 5.69±2.37 | 5.48±1.74 | 70.01±19.43 |
| **Cultural values** | 202 | | 12.31±5.38 | 15.22±5.18 | 10.45±3.65 | 7.03±2.67 | 4.43±2.94 | 11.69±4.76 | 6.04±2.34 | 5.66±1.23 | 73.33±19.54 |
| **Others** | 34 | | 11.83±5.17 | 14.34±5.65 | 10.08±3.87 | 6.63±2.98 | 4.24±2.87 | 11.43±3.89 | 5.66±2.95 | 5.43±1.97 | 69.67±19.33 |
| **F** | | | 1.122 | 2.150 | 2.589 | 2.623 | -2.328 | -2.121 | -1.780 | -.862 | -1.714 |
| **p** | | | .262 | ***.003*** | .245 | ***.001*** | ***.020*** | ***.033*** | .072 | .064 | .724 |
| **Bonferroni** | | | - | 1>3>2>4 | - | 1>3>2>4 | 3>2>4>1 | 1>3>2>4 | - | - | - |
| **Previous condom use** |  |  |  |  |  |  |  |  |  |  |  |
| **Yes** | 411 | | 11.78±4.46 | 14.52±5.71 | 10.18±3.87 | 6.72±2.75 | 4.30±2.21 | 11.62±4.54 | 5.94±2.49 | 5.52±1.71 | 70.63±20.36 |
| **No** | 542 | | 11.98±4.66 | 14.46±5.48 | 10.09±3.59 | 6.66±2.45 | 4.24±2.16 | 11.34±4.49 | 5.53±2.27 | 5.48±2.45 | 69.82±18.75 |
| **t** | | | -2.333 | -.662 | .-546 | -.593 | -3.333 | .249 | -.120 | -1.456 | -1.122 |
| **p** | | | ***.002*** | .430 | .210 | .630 | ***.002*** | .805 | .564 | .654 | .270 |
| **Why choose a condom** | | | | | | | | | | | |
| **Easy to reach ^(1)^** | 256 | | 12.32±6.78 | 16.50±5.63 | 11.35±3.79 | 7.67±2.76 | 5.64±2.24 | 12.43±3.89 | 6.45±2.78 | 5.92±1.56 | 75.39±19.76 |
| **Easy of use ^(2)^** | 149 | | 12.88±5.54 | 15.53±5.64 | 11.09±3.81 | 7.68±2.68 | 5.37±2.37 | 12.65±4.73 | 6.87±2.73 | 5.48±1.74 | 70.01±19.43 |
| **No side effects ^(3)^** | 409 | | 12.31±5.65 | 15.42±5.64 | 11.45±3.98 | 7.03±2.67 | 5.43±2.43 | 12.74±4.86 | 6.04±2.76 | 5.66±1.89 | 73.33±19.56 |
| **Others^(4)^** | 139 | | 11.83±6.45 | 14.34±5.67 | 10.08±3.67 | 6.63±2.87 | 5.24±2.65 | 11.73±4.65 | 5.78±2.98 | 5.43±2.01 | 70.67±19.68 |
| **F** | | | 1.122 | 2.150 | 2.589 | 2.623 | -2.328 | -2.121 | -1.780 | -.862 | -1.714 |
| **p** | | | .262 | ***.003*** | .245 | ***.001*** | ***.002*** | ***.001*** | ***.032*** | .064 | .724 |
| **Bonferroni** | | | - | 1>2>3>4 | - | 2>1>3>4 | 1>3>2>4 | 3>2>1>4 | 2>1>3>4 | - | - |
| **Reason to choose a retraction** | | | | | | | | | | | |
| **Easy of use ^(1)^** | 698 | | 12.20±4.21 | 14.64±5.45 | 10.58±4.37 | 6.79±2.21 | 6.79±2.21 | 4.79±2.15 | 11.23±4.32 | 5.58±2.18 | 71.26±18.11 |
| **No side effects ^(2)^** | 198 | | 11.88±4.32 | 14.48±4.98 | 10.11±4.56 | 6.68±2.60 | 6.68±2.60 | 4.25±2.18 | 11.47±4.52 | 5.71±2.38 | 70.13±19.13 |
| **Others ^(3)^** | 57 | | 11.95±4.17 | 13.75±4.65 | 10.23±3.76 | 5.65±2.31 | 5.95±2.91 | 4.48±2.45 | 10.53±4.62 | 6.48±2.38 | 70.26±18.43 |
| **F** | | | -2.333 | -.662 | .-546 | -.593 | -3.333 | .249 | -.120 | -1.456 | -1.122 |
| **p** | | | ***.002*** | .430 | .210 | .630 | ***.002*** | .805 | .564 | .654 | .270 |
| **Bonferroni** | | | **1>2>3** | **-** | - | **-** | 1>2>3 | - | **-** | **-** | **-** |

***FP: Family Planning, SMS*: Sexual Myhts Scale, t: t test, F: Anova Test***

**Alt Text:** Supplementary file 1 compares family planning characteristics among men (n=953) and their scores on the Sexual Myths Scale (SMS) across various dimensions. It displays mean ± standard deviation for sexual orientation, gender, age and sexuality, sexual behavior, masturbation, sexual violence, sexual satisfaction, and total SMS scores based on family planning usage and methods. Statistical significance is indicated by t and F values with corresponding p-values, including Bonferroni post hoc results for significant differences among groups. Asterisks highlight significant findings (p < 0.05).

**Supplementary file 2. Comparison of Men's Value Judgments about Sexuality and SMS and Sub-Dimensions (n=953)**

| **Variables** | **n** | **Sexual Orientation*** | **Gender*** | **Age and Sexuality*** | **Sexual Behavior*** | **Mastürbation*** | **Sexual Violence *** | **Sex *** | **Sexual Satisfaction*** | **SMS* Total Puan Average** |
| --- | --- | --- | --- | --- | --- | --- | --- | --- | --- | --- |
| **Sexual intercourse before marriage** | | | | | | | | | | |
| **Yes** | 484 | 11.88±4.32 | 14.4875±4.98 | 10.11±4.56 | 6.68±2.60 | 6.68±2.60 | 4.25±2.18 | 11.47±4.52 | 5.71±2.38 | 70.13±19.13 |
| **No** | 469 | 11.95±4.17 | 13.75±4.65 | 10.23±3.76 | 5.65±2.31 | 5.95±2.91 | 4.48±2.45 | 10.53±4.62 | 6.48±2.38 | 70.26±18.43 |
| **t** | | 2.482 | 72.063 | 42.379 | 52.816 | 2.302 | 106.177 | 9.276 | 42.705 | 57.568 |
| **p** | | **.030** | **.000** | **.000** | **.000** | **.043** | **.000** | **.000** | **.000** | **.000** |
| **Regular sexual intercourse** | | | | | | | | | | |
| **Yes** | 569 | 12.62±1.92 | 11.25±4.43 | 7.75±2.43 | 5.12±1.80 | 4.00±2.07 | 8.12±2.85 | 4.87±1.80 | 5.00±1.11 | 58.75±12.11 |
| **No** | 384 | 13.03±5.91 | 10.46±5.12 | 8.09±3.64 | 5.04±2.43 | 4.08±2.03 | 7.66±4.04 | 4.79±2.41 | 4.45±2.01 | 57.63±20.86 |
| **t** | | ***-2.357*** | ***-2.344*** | -2.564 | -.914 | ***-3.240*** | .513 | ***3.299*** | -1.122 | -1.561 |
| **p** | | ***.003*** | ***.000*** | .270 | .368 | ***.005*** | .577 | ***.005*** | .270 | .119 |
| **Prejudice about sexuality** | | | | | | | | | | |
| **Yes** | 89 | 11.54±4.34 | 16.76±6.01 | 11.62±4.16 | 7.66±2.954 | 4.97±2.87 | 12.64±4.05 | 5.78±2.52 | 5.94±1.89 | 75.29±21.93 |
| **No** | 864 | 11.56±4.42 | 16.34±5.41 | 11.23± 4.10 | 8.95±2.76 | 4.78±2.76 | 13.77±2.92 | 5.98±2.86 | 6.43±1.76 | 78.87±15.39 |
| **t** | | 1.482 | 72.063 | 42.379 | 22.816 | 2.302 | 76.177 | 6.276 | 32.875 | 78.568 |
| **p** | | ***.045*** | ***.005*** | .765 | ***.000*** | ***.031*** | ***.000*** | ***.000*** | ***.000*** | ***.000*** |
| **Men and women are not equal sexually** | | | | | | | | | | |
| **Yes** | 535 | 12.56±4.62 | 14.48±5.61 | 11.18±3.98 | 6.98±2.63 | 4.2791±2.20 | 11.37±4.51 | 5.89±2.34 | 6.54±1.84 | 75.38±19.54 |
| **No** | 418 | 11.54±4.79 | 14.38±5.87 | 9.83±3.79 | 6.44±2.40 | 4.38±2.87 | 11.76±4.51 | 5.58±2.56 | 6.56±1.89 | 72.17±19.49 |
| **t** | | 2.482 | 72.063 | 42.379 | 52.816 | 2.302 | 106.177 | 9.276 | 42.705 | 73.6268 |
| **p** | | ***.030*** | ***.000*** | ***.000*** | ***.000*** | ***.043*** | ***.000*** | ***.000*** | ***.042*** | ***.213*** |
| **Virginity is important for the woman** | | | | | | | | | | |
| **Yes** | 801 | 12.79±4.58 | 14.82±5.26 | 11.286±3.46 | 7.39±2.43 | 4.79±1.98 | 11.64±4.57 | 6.13±2.47 | 4.66±1.73 | 72.54±19.44 |
| **No** | 152 | 11.99±4.69 | 15.85±5.10 | 11.11±3.50 | 7.26±2.46 | 4.24±2.20 | 12.67±3.91 | 6.00±2.34 | 5.87±1.62 | 71.94±17.21 |
| **t** | | .677 | 1.168 | 2.564 | 1.872 | 2.640 | 1.413 | .498 | 3.674 | 1.784 |
| **p** | | .218 | ***.000*** | ***.000*** | ***.001*** | *.*213 | ***.040*** | *.*231 | ***.000*** | ***.000*** |
| **Sexual fantasies are not conforming to beliefs** | | | | | | | | | | |
| **Yes** | 312 | 12.09±4.88 | 11.22±5.269 | 8.28±3.46 | 5.39±2.43 | 4.09±1.98 | 8.64±4.47 | 5.13±2.37 | 4.66±1.73 | 59.54±19.44 |
| **No** | 641 | 11.95±4.63 | 13.96±5.71 | 9.86± 3.811 | 6.47±2.62 | 4.18±2,51 | 10.99±4.68 | 5.67±2.42 | 5.44 ±1.83 | 68.56±19.98 |
| **t** | | .531 | -4.134 | -3.437 | -3.866 | -1.937 | -4.737 | .724 | -1.440 | -3.770 |
| **p** | | .593 | ***.000*** | ***.000*** | ***.000*** | ,057 | ***.000*** | .469 | ***.005*** | ***.000*** |
| **Women don't need to enjoy sexually** | | | | | | | | | | |
| **Yes** | 641 | 11.58±4.276 | 16.53±4.80 | 10.86±3.472 | 7.44±2.33 | 4.58±2.41 | 13.15±3.72 | 5.96±2.30 | 5.98±1.50 | 76.11±17.34 |
| **No** | 311 | 11.91±4.494 | 15.85±5.10 | 11.11±3.508 | 7.26±2.46 | 4.24±2.20 | 12.67±3.91 | 6.00±2.34 | 5.87±1.62 | 74.94±17.21 |
| **t** | | .808 | 96.365 | 65.140 | 66.844 | 3.237 | 112.175 | 14.085 | 61.286 | 80.796 |
| **p** | | .446 | ***.000*** | ***.000*** | ***.000*** | ***.040*** | ***.000*** | ***.000*** | ***.000*** | ***.000*** |
| **Sexual intercourse and its time should be determined by the man** | | | | | | | | | | |
| **Yes** | 286 | 12.01±4.43 | 15.70±4.85 | 10.83±3.50 | 7.31±2.45 | 4.40±2.21 | 12.55±3.84 | 5.85±2.35 | 5.83±1.63 | 74.50±17.42 |
| **No** | 667 | 11.84±4.63 | 13.97±5.79 | 9.83±3.76 | 6.42±2.60 | 4.21±2.17 | 11.00±4.69 | 5.65±2.38 | 5.36±1.75 | 68.32±19.99 |
| **t** | | .647 | 4.167 | 3.961 | 4.999 | 1.180 | 5.313 | 1.198 | 3.944 | 4.784 |
| **p** | | .618 | ***.000*** | ***.000*** | ***.000*** | ***.000*** | ***.000*** | *.*231 | ***.000*** | ***.000*** |
| **In sexual intercourse, what the man wants is obeyed** | | | | | | | | | | |
| **Yes** | 398 | 12.09±4.884 | 11.22±5.269 | 8.28±3.46 | 5.39±2.43 | 4.09±1.98 | 8.64±4.47 | 5.13±2.37 | 4.66±1.73 | 59.54±19.44 |
| **No** | 555 | 11.95±4.63 | 13.96±5.71 | 9.86± 3.81 | 6.47±2.62 | 4.18±2.51 | 10.99±4.68 | 5.67±2.42 | 5.44 ±1.83 | 68.56±19.98 |
| **t** | | .531 | -4.134 | -3.437 | -3.866 | -1.937 | -4.737 | .724 | -1.440 | -3.770 |
| **p** | | .593 | ***.000*** | ***.000*** | ***.000*** | .057 | ***.000*** | .469 | .150 | ***.000*** |
| **Society has a bias against sexually transmitted diseases** | | | | | | | | | | |
| **Yes** | 201 | 12.25±5.581 | 16.50±5.50 | 11.25±3.59 | 7.07±2.66 | 4.21±2.04 | 12.00±3.79 | 6.17±2.65 | 5.92±1.56 | 75.39±19.76 |
| **No** | 752 | 11.88±4.54 | 14.43±5.57 | 10.09±3.71 | 6.68±2.58 | 4.27±2.19 | 11.45±4.53 | 5.69±2.37 | 5.48±1.74 | 70.01±19.43 |
| **t** | | .677 | 1.168 | 2.564 | 1.872 | 2.640 | 1.413 | .498 | 3.674 | 1.784 |
| **p** | | .218 | ***.000*** | ***.000*** | ***.001*** | *.*276 | ***.040*** | .231 | ***.000*** | ***.000*** |
| **Having sexual intercourse during pregnancy is a sin** | | | | | | | | | | |
| **Yes** | 441 | 10.33±3.69 | 14.00±5.48 | 9.73±3.34 | 6.43±2.43 | 3.26±1.65 | 11.70±5.18 | 5.66±2.18 | 5.20±1.84 | 66.33±19.02 |
| **No** | 512 | 11.94±4.59 | 14.50±5.58 | 10.14±3.72 | 6.70±2.59 | 4.30±2.19 | 11.46±4.49 | 5.71±5.51 | 5.51±1.73 | 70.29±19.46 |
| **t** | | ***-1.904*** | -.492 | -.598 | -.593 | ***-3.333*** | .285 | -.111 | -.970 | -1.099 |
| **p** | | ***.026*** | .623 | .550 | .577 | ***.001*** | .776 | .920 | .332 | .270 |
| **Having sex while menstruating is a sin** | | | | | | | | | | |
| **Yes** | 897 | 11.78±3.56 | 13.96±5.63 | 10.53±3.46 | 6.31±2.97 | 3.86±1.15 | 11.89±4.97 | 5.23±2.54 | 5.68±3.49 | 66.65±19.02 |
| **No** | 56 | 10.24±3.12 | 13.41±5.14 | 9.21±2.98 | 6.11±2.41 | 3.29±1.31 | 10.76±3.98 | 5.31±3.32 | 5.74±2.18 | 70.54±19.46 |
| **t** | | -2.333 | -.662 | .-546 | -.381 | -2.871 | .129 | -.120 | -1.456 | -1.122 |
| **p** | | **.002**** | .430 | .210 | .630 | **.002** | .089 | .564 | .654 | .270 |
| **Having sexual intercourse in menopause is a sin** | | | | | | | | | | |
| **Yes** | 206 | 11.58±4.27 | 16.53±4.807 | 10.86±3.47 | 7.44±2.33 | 4.58±2.41 | 13.15±3.72 | 5.96±2.30 | 5.98±1.50 | 76.11±17.34 |
| **No** | 747 | 11.91±4.49 | 15.85±5.103 | 11.11±3.50 | 7.26±2.46 | 4.24±2.20 | 12.67±3.91 | 6.00±2.34 | 5.87±1.62 | 74.94±17.21 |
| **t** | | .808 | 96.365 | 65.140 | 66.844 | 3.237 | 112.175 | 14.085 | 61.286 | 80.796 |
| **p** | | .446 | ***.000*** | ***.000*** | ***.000*** | ***.040*** | ***.000*** | ***.000*** | ***.000*** | ***.000*** |
| **Different sexual identities are sins** | | | | | | | | | | |
| **Yes** | 286 | 12.01±4.43 | 15.70±4.85 | 10.83±3.50 | 7.31±2.45 | 4.40±2.21 | 12.55±3.84 | 5.85±2.35 | 5.83±1.63 | 74.50±17.42 |
| **No** | 667 | 11.84±4.63 | 13.97±5.79 | 9.83±3.76 | 6.42±2.60 | 4.21±2.17 | 11.00±4.69 | 5.65±2.38 | 5.36±1.75 | 68.32±19.99 |
| **t** | | .647 | 4.167 | 3.961 | 4.999 | 1.180 | 5.313 | 1.198 | 3.944 | 4.784 |
| **p** | | .618 | ***.000*** | ***.000*** | ***.000*** | ***.000*** | ***.000*** | *.*231 | ***.000*** | ***.000*** |

***SMS*: Sexual Myhts Scale, t: t test,***

**Alt Text:** Supplementary file 2 presents a comparison of men’s value judgments about sexuality and their scores on the Sexual Myths Scale (SMS) (n=953). It includes mean ± standard deviation for various dimensions such as sexual orientation, gender, age and sexuality, sexual behavior, masturbation, sexual violence, sexual satisfaction, and total SMS scores based on beliefs about sexual intercourse before marriage, regular sexual intercourse, sexual prejudice, and attitudes toward women’s sexual roles. Statistical significance is indicated by t values and corresponding p-values, with asterisks denoting significant findings (p < 0.05).
