# Supplementary material for: Exploring sexual myths and influencing factors among Muslim men in Turkey: a cross-sectional analysis
Source: Basic Clin Androl. 2025 Dec 3;35:46. doi: 10.1186/s12610-025-00296-9 (PMC12673704; doi:10.1186/s12610-025-00296-9)
Supplement: Supplementary file 2 — Supplementary Material 2 [file 12610_2025_296_MOESM2_ESM.docx]

# Supplementary File 1. Family Planning Attitude and Behavior Inventory

This inventory was developed by the researchers based on relevant literature [6,9]. It consists of seven items related to the use of family planning methods, reasons for preferences, and beliefs about these methods. Response options are presented as Yes/No or categorical choices.

Items:

- 1. Do you currently use any family planning (FP) method? (Yes/No)
- 2. Which FP method do you use? (Condom / Vasectomy / Withdrawal / Other)
- 3. What is the main reason for using FP? (Not having children / Other reasons)
- 4. Do you consider your sexual life comfortable with your FP method? (Yes/No)
- 5. What is the main reason for not using FP? (Beliefs / Societal pressure / Cultural values / Other)
- 6. Have you ever used a condom before? (Yes/No)
- 7. Why did you choose a condom or withdrawal method? (Easy to reach / Easy to use / No side effects / Other)
