# Supplementary material for: Exploring sexual myths and influencing factors among Muslim men in Turkey: a cross-sectional analysis
Source: Basic Clin Androl. 2025 Dec 3;35:46. doi: 10.1186/s12610-025-00296-9 (PMC12673704; doi:10.1186/s12610-025-00296-9)
Supplement: Supplementary file 3 — Supplementary Material 3 [file 12610_2025_296_MOESM3_ESM.docx]

# Supplementary File 2. Inventory of Sexual Value Judgments

This inventory was developed by the researchers based on relevant literature [6,9,12]. It contains 16 items that assess participants’ value judgments and religious beliefs regarding sexuality. Response options are Yes/No.

Items:

- 1. Do you believe sexual intercourse before marriage is acceptable? (Yes/No)
- 2. Do you believe regular sexual intercourse is necessary in a relationship? (Yes/No)
- 3. Do you think society holds specific sexual value judgments regarding sexuality? (Yes/No)
- 4. Do you believe men and women are not equal sexually? (Yes/No)
- 5. Do you believe virginity is important for women? (Yes/No)
- 6. Do you believe sexual fantasies contradict religious or cultural beliefs? (Yes/No)
- 7. Do you believe women do not need to enjoy sexual activity? (Yes/No)
- 8. Should the timing of sexual intercourse be determined by the man? (Yes/No)
- 9. In sexual intercourse, should the man’s desire always be obeyed? (Yes/No)
- 10. Do you believe society has prejudice against sexually transmitted diseases? (Yes/No)
- 11. Is having sexual intercourse during pregnancy a sin? (Yes/No)
- 12. Is having sexual intercourse while menstruating a sin? (Yes/No)
- 13. Is having sexual intercourse during menopause a sin? (Yes/No)
- 14. Are different sexual identities considered sinful? (Yes/No)
- 15. Do you believe sexual intercourse should only serve reproductive purposes? (Yes/No)
- 16. Do you believe men should always initiate sexual intercourse? (Yes/No)
